# Computational Imaging Prediction of Starburst-Effect Diffraction Spikes

**DOI:** 10.1038/s41598-018-34400-z

**Published:** 2018-11-16

**Authors:** Markus Lendermann, Joel Shi Quan Tan, Jin Ming Koh, Kang Hao Cheong

**Affiliations:** 1 National University of Singapore High School of Mathematics and Science, 20 Clementi Avenue 1, S129957 Singapore, Singapore; 2 Yong Loo Lin School of Medicine, National University of Singapore, S119228 Singapore, Singapore; 3 Engineering Cluster, Singapore Institute of Technology, 10 Dover Drive, S138683 Singapore, Singapore

## Abstract

When imaging bright light sources, rays of light emanating from their centres are commonly observed; this ubiquitous phenomenon is known as the starburst effect. The prediction and characterization of starburst patterns formed by extended sources have been neglected to date. In the present study, we propose a novel trichromatic computational framework to calculate the image of a scene viewed through an imaging system with arbitrary focus and aperture geometry. Diffractive light transport, imaging sensor behaviour, and implicit image adjustments typical in modern imaging equipment are modelled. Characterization methods for key optical parameters of imaging systems are also examined. Extensive comparisons between theoretical and experimental results reveal excellent prediction quality for both focused and defocused systems.

## Introduction

Captured images of light sources commonly exhibit the *starburst effect*, an optical phenomenon comprising apparent rays of light emanating from their centres. These rays, known as *diffraction spikes*, are also observable by the naked human eye, usually at night. Diffraction spikes in telescope images of stars and other illuminated bodies^1–3^ introduce uncertainties in luminosity-dependent measurements, but can be useful in localization techniques^4^. The phenomenon occurs on all light sources and affects a wide range of imaging systems, including photography^5–7^, medical endoscopy^8^, and telemetry acquisition systems^9^, with higher-intensity sources yielding more prominent spikes.

Often accompanied with lens flare^10,11^, the starburst effect arises due to the diffraction of light as it propagates past the limiting aperture of the imaging system^12^. A Fourier optics formulation is typically employed, where the diffraction-limited point spread function is given by the Fourier transform of the exit pupil shape. It is common for imaging systems at high *f*-numbers to have polygonal apertures—these admit high spatial frequency components along axes perpendicular to the polygonal edges^13,14^, hence forming the perceived spikes. In reflective telescopes, the support vanes of secondary mirrors result in a diffraction pattern similar to that formed by multiple intersecting slits^15^.

The suppression and intensification of the starburst effect have received much attention to date. Efforts have been made to render visually similar effects in image post-processing^6^ and minimize diffraction artifacts in high dynamic range (HDR) photography^5^. In astronomy, software modelling Bahtinov masks and spider-diffraction have been developed^16^, and the reduction of diffractive effects on segmented mirrors is crucial for telescope design^17^. Simulation toolsets and methods are also available for astronomical imagery, encompassing light transport effects including weak gravitational lensing and Doppler shift^18–20^. Outside of astronomy, however, limited attention has been placed on correctly predicting the images of extended objects on general apertures and focus, with the optical parameters and implicit image processing of the imaging system taken into account; a lacuna in this discipline therefore remains. While predicting the corresponding image of a point-like object entails a calculation of the point-spread function (PSF), predictions for extended objects require a convolution of the PSF with the object field, significantly increasing the complexity of the problem. Addressing this gap enables greater accuracy and generality in modelling the starburst effect, thereby enhancing its diverse applications, especially in image prediction on commercial imaging systems—such is the focus of our study.

This paper presents a rigorous framework for calculating the image of a scene viewed through an imaging system with arbitrary focus. A Fourier optics formulation is first discussed, followed by a computational image prediction framework. The characterization of a benchmark imaging system and the adopted experimental method are then described, with extensive comparisons between theoretical and experimental results.

## Optics Formulation

In Fourier optics, a system of lenses and apertures can be reduced to corresponding entrance and exit pupils, wherein diffractive effects can be equivalently treated^21^; the exit pupil is used throughout this paper. The media of the object and image spaces are taken to be identical, therefore the nodal points and principal planes are coincident^22^. A plausible alternative to the adopted Fourier-optical formulation is Monte Carlo ray-tracing extended to model edge diffraction^23^, though this remains outside the scope of the current study.

**Figure 1:**
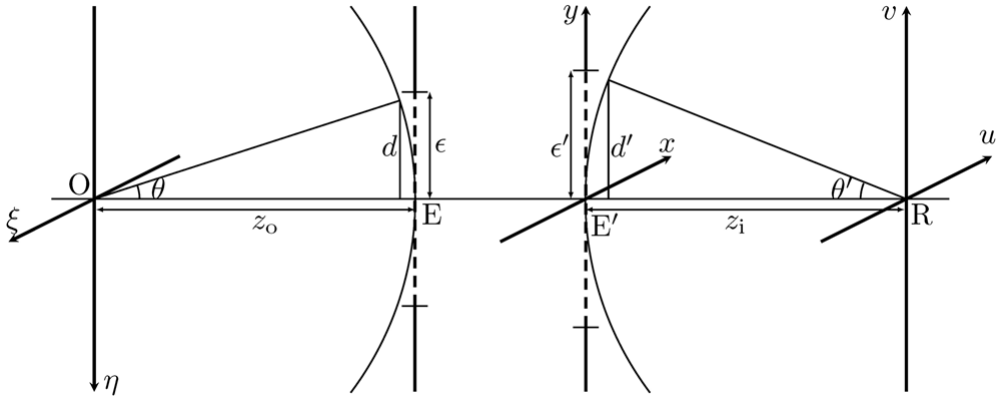
O, R, object and image planes; E, E’, entrance and exit pupils with reference lengths *ε*, *ε*′ respectively.

The system geometry is defined in Fig. 1. The geometrical coordinates on the image plane R are denoted (*u*, *v*). The heights *d* and *d*′ are defined at the entrance and exit pupils, as shown by the intersection of the limiting rays with reference spheres centered at the origins of the object and image planes respectively. The linear magnification of the system is then $M=d{z}_{{\rm{i}}}/d^{\prime} {{z}}_{{\rm{o}}}$, where *z*_o_ and *z*_i_ are the distances between the object plane and entrance pupil, and between the image plane and the exit pupil, respectively.

Here an extended object at O is illuminated by spatially incoherent light. The actual image intensity distribution can be written ${I}_{i}(u,v)=|h(u,v{)|}^{2}\otimes {I}_{g}(u,v)$, where $\otimes$ denotes a convolution, *I*_*g*_ is the magnified object-space intensity distribution, and $|h{|}^{2}$ and *h* are the intensity and amplitude point spread functions respectively. Note that *h* is complex, encoding both amplitude and phase information. To compute the convolution, the optical transfer function (OTF) ${\mathcal H} = {\mathcal F} \{|h{|}^{2}\}$ given by

1 $$ {\mathcal H} ({f}_{u},{f}_{v})=\frac{{ {\mathcal F} }^{-1}\{| {\mathcal F} \{H{\}|}^{2}\}}{\int {\int }_{-\infty }^{\infty }| {\mathcal F} \{H\}(u,v{)|}^{2}\,{\rm{d}}u\,{\rm{d}}v}$$

is utilized, where $H= {\mathcal F} \{h\}$ is the amplitude transfer function (ATF) and ${\mathcal F} \{\cdot \}$ denotes the two-dimensional Fourier transform operator. Calculation of the OTF from a known ATF in this manner is known as the *double-transform* method^24^. The ATF of an imaging system takes the form

2 $$H({f}_{u},{f}_{v})=P(\lambda {z}_{i}\,{f}_{u},\,\lambda {z}_{i}\,{f}_{v}){{\rm{e}}}^{\iota kW(\lambda {z}_{i}{f}_{u},\lambda {z}_{i}{f}_{v})},$$

where $k=2\pi /\lambda$ and *P*(*x*, *y*) is the exit pupil function describing the bounded pupil area ${{\mathscr{S}}}_{{\rm{p}}{\rm{u}}{\rm{p}}}$. The exponent in Equation (2) accounts for any phase shift *kW*(*x*, *y*) at the exit pupil due to aberrations. Considering on-axis image points (see Fig. A.1 of Supplementary Information), *kW*(*x*, *y*) can be obtained by subtracting the ideal phase distribution across the exit pupil from the actual one. Therefore,

3 $$W(x,y)=\frac{1}{2}(\frac{1}{{z}_{i}}-\frac{1}{{z}_{i}+{\rm{\Delta }}z})({x}^{2}+{y}^{2}),$$

where Δ*z* is the distance between the in-focus image plane F and the out-of-focus image plane R. These calculations are valid in the paraxial approximation, an inherent limitation in Fourier-optical formulations.

For simple pupil shapes, the OTF can be solved analytically from Equation (1) with the normalized autocorrelation function of *H* ^21,25^. For complex pupil shapes, either the double-transform method or normalized autocorrelation may be performed numerically. While the former relies on fast Fourier transform (FFT) algorithms, the latter requires a polygon clipping algorithm^26^ for each sampling point of the OTF, to determine the area over which $\exp [\iota kW(x,y)]$, a highly oscillatory function at large defocusing, may be numerically integrated. Such a procedure is both time and memory intensive; the double-transform method is hence preferable.

The isoplanacity of the imaging lens system is assumed in this model as a simplification. A more complete treatment will entail the characterization of the modulation transfer function (MTF) of the lens system over the image area; such a method, however, yields a spatially variant PSF that is incompatible with a Fourier-optical formulation. To retain the low computational cost of Fourier-optical approaches, the imaging region of interest is taken to lie within the isoplanatic patch of the lens. This condition is expected to be satisfied if the paraxial condition holds.

## Computational Model

**Figure 2:**
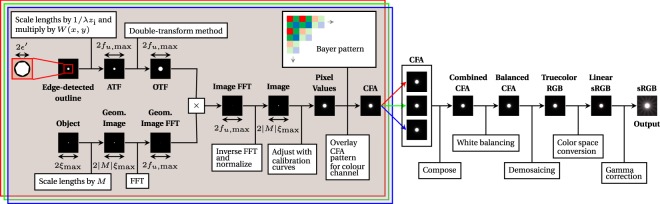
Flowchart illustrating the image prediction framework. The process comprises two sequential segments—the double-transform method first computes the channel-specific pixel values, followed by a postprocessing pipeline reflecting image adjustments standard on modern imaging equipment. Note that thumbnails from "Pixel Values" onwards are cropped and expanded for clarity.

In this section, the computational framework for the prediction of diffraction spikes is discussed. The process is divided into two primary segments (Fig. 2). First, the double-transform method computes the theoretical channel-specific raw pixel values; a post-processing pipeline then renders the final colour image. In this manner, the predicted colour image *corresponds directly* with images taken by imaging equipment, and the two can therefore be compared.

A trichromatic approach is adopted—predictions are made based on the composition of three monochromatic colour channels, each of a specific peak wavelength. This approximation yields a significant reduction in computational complexity, as compared to a polychromatic approach involving integration across the bandpasses of imaging sensor elements. In the adopted approach, the wavelength-dependent sensitivity of the sensor is simplistically treated via empirical characterization on each channel, as will be described in the next section; this implies that the full spectral power distribution of the source need not be known. A trichromatic approach may be analogous to the technological nature of modern imaging sensors and displays^27^, and to biological vision in humans and many other organisms^28–30^.

### Raw Pixel Data Prediction

We seek to calculate the theoretical intensity distribution across the imaging sensor, such that the raw pixel values can be computed. First, the normalized object intensity ${I}_{o}\in \mathrm{[0,}\,\mathrm{1]}$ is represented by an *m* × *n* matrix **A** covering a geometrical size of $2{\xi }_{{\rm{\max }}}\times 2{\eta }_{{\rm{\max }}}$. The geometrical image therefore has a size $\mathrm{2|}M|{\xi }_{{\rm{\max }}}\times \mathrm{2|}M|{\eta }_{{\rm{\max }}}$ and a normalized intensity matrix **B** given by ${{\bf{B}}}_{i,j}={{\bf{A}}}_{isgnM,jsgnM}$. To calculate the OTF matrix ${\boldsymbol{ {\mathcal H} }}$, the pupil function matrix in the frequency domain ${\boldsymbol{\mathcal P}}$ is first constructed by scaling ${{\mathscr{S}}}_{{\rm{p}}{\rm{u}}{\rm{p}}}$ (defined in the spatial domain) by 1/*λz*_*i*_, and uniformly sampling it *m* × *n* times within the domain of ${\boldsymbol{ {\mathcal B} }}= {\mathcal F} \{{\bf{B}}\}$. The ATF matrix is then

4 $${{\bf{H}}}_{i,j}={{\boldsymbol{\mathcal P}}}_{i,j}\,\exp \{\frac{\iota k{\lambda }^{2}{z}_{{\rm{i}}}^{2}}{2}(\frac{1}{{z}_{i}}-\frac{1}{{z}_{i}+{\rm{\Delta }}z})\times [{({f}_{u,\max }\frac{m-2i}{m})}^{2}+{({f}_{v,\max }\frac{n-2j}{n})}^{2}]\},$$

where ${f}_{u,{\rm{\max }}}=m\mathrm{/4|}M|{\xi }_{{\rm{\max }}}$ and ${f}_{v,{\rm{\max }}}=n\mathrm{/4|}M|{\eta }_{{\rm{\max }}}$ are the Nyquist frequencies along the *u* and *v* axes. The image must be of sufficiently high resolution such that the Nyquist frequency is larger than the cut-off frequency of the ATF. Sufficient null padding is also necessary for periodicity breaking. Utilizing the double-transform method, the OTF matrix ${\boldsymbol{ {\mathcal H} }}$ can be computed as ${\boldsymbol{ {\mathcal H} }}= {\mathcal F} \{{| {\mathcal F} }^{-1}\{{\bf{H}}{\}|}^{2}\}$. The predicted image intensity distribution is then ${\bf{C}}={ {\mathcal F} }^{-1}\{{\boldsymbol{ {\mathcal H} }}\,\circ \,{\boldsymbol{ {\mathcal B} }}\}$ where $\circ$ denotes the Hadamard product of matrices.

The actual intensity distribution incident on the imaging sensor is therefore *κ***C**, where *κ* is a proportionality constant dependent on the system geometry. The raw pixel value matrix **D** is then

5 $${{\bf{D}}}_{i,j}={\mathscr{Z}}(\kappa {{\bf{C}}}_{i,j}t/{\varphi }_{0}),$$

where *t* is the exposure time, ${\varphi }_{0}$ is a reference radiant exposure for normalization, and ${\mathscr{Z}}$ is the sensor response function. The form of ${\mathscr{Z}}$ is intrinsic upon the physical construction of the sensor, and ${\varphi }_{0}$ is specific for a given source and sensor configuration. The physical parameters which ${\mathscr{Z}}$ and ${\varphi }_{0}$ depend on are detailed in Supplementary Information B, alongside characterization methods in the next section.

Finally, an appropriate colour filter is applied to **D**, yielding the predicted channel-specific pixel values. The colour filter is dependent upon the construction of the imaging sensor, and can be represented by a Bayer pattern. This entire process is repeated for all three colour channels to yield a complete pixel-wise prediction in colour filter array (CFA) form.

### Post-Processing Rendering

Further stages of processing are required to apply various adjustments that are oftentimes implicit in modern imaging equipment. In the un-demosaiced CFA form, white balancing is performed, followed by the execution of a demosaicing algorithm to yield a true colour RGB image^31^. Colour space conversion is then applied for accurate display on a computer monitor or in print^32^. Gamma correction may also be applied. The final result from this process is a predicted colour image that corresponds directly to one captured by a modern imaging system, computed from first principles—the excellent accuracy of this method is demonstrated in later sections.

## Imaging System Characterization

Predicting the behaviour of an imaging system through the presented computational model requires several characteristic parameters of the system to be known. These include the linear magnification of the lens system and the positions and sizes of the entrance and exit pupils. The positions of the principal planes are also necessary to ascertain the lens geometry at different focusing distances. As a demonstration, a Nikon D7200 DSLR camera with an AF Nikkor 50 mm *f*/1.8D prime lens is used as a verification benchmark. However, it is worth noting that the model presented in this paper is applicable to arbitrary imaging systems in general.

### Pupil & Principal Plane Locations

Characteristic data for the examined lens system is available in existing literature^33^. The extracted pupil and principal plane positions relative to the sensor plane are presented in Table B.1 of the Supplementary Information. While these measurements are valid for the camera at infinity focus, changing the focusing distance of the lens will result in a shift of the pupil locations. This can be calculated by treating the compound camera lens as a single thin lens with the principal planes^12^.

### Focus Adjustment

**Figure 3:**
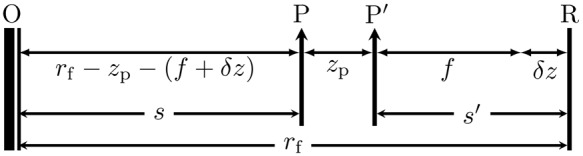
Geometric schematic of a prime lens system focused at a distance *r*_f_ away from the sensor plane R.

The *effective focal length* (EFL) of the system is denoted *f*. Consider the distance between the front principal plane and the object plane on which the system is focused (hereinafter *s*, see Fig. 3), and the distance between the rear principal plane and the sensor plane (hereinafter $s^{\prime}$). Clearly $s^{\prime} =f$ when the lens is focused at infinity; thus, from Table B.1, *f* = (51.5 ± 0.1) mm.

The examined system is equipped with a *prime lens*—that is, focusing is achieved by the simultaneous movement of all lens elements back and forth within the lens enclosure as a single system. To achieve a finite focusing distance *r*_f_ away from the sensor plane, the lens elements must shift forward by a distance $\delta z$ (Fig. 3). Denote the distance between P and P′ as *z*_p_. Then, $s={r}_{{\rm{f}}}-{z}_{{\rm{p}}}-s^{\prime}$ and $s^{\prime} =f+\delta z$. Therefore,

6 $$\frac{1}{f}=\frac{1}{{r}_{{\rm{f}}}-{z}_{{\rm{p}}}-(f+\delta z)}+\frac{1}{f+\delta z},$$

from which *δz* and Δ*z* as defined in Fig. A.1 can be calculated for path-length error computation (see Supplementary Information A). This characterization method can be applied to prime lens systems in general. Zoom lenses are more complex due to their adjustable focal lengths; more detailed specifications are necessary for a complete characterization.

### Pupil Sizes

The pupil sizes *d* and *d*′ remain to be determined. The geometry of Fig. 1 indicates $d=\varepsilon {z}_{{\rm{o}}}/({\varepsilon }^{2}+{z}_{{\rm{o}}}^{2}{)}^{\mathrm{1/2}}$ and $d^{\prime} =\varepsilon ^{\prime} {z}_{{\rm{i}}}/({\varepsilon ^{\prime} }^{2}+{z}_{{\rm{i}}}^{2}{)}^{\mathrm{1/2}}$, where *ε* and *ε*′ are the *reference length scales* measured from the optical axis to the farthest point on the pupil. To measure *ε* and *ε*′, the lens was detached from the camera body, and a second camera was used to photograph the entrance and exit pupils. The shapes and sizes of the pupils were determined using computational edge-detection (see Fig. B.1 of the Supplementary Information), on which ${{\mathscr{S}}}_{{\rm{pup}}}$, *ε* and *ε*′ can be defined.

### Sensor Response

**Figure 4:**
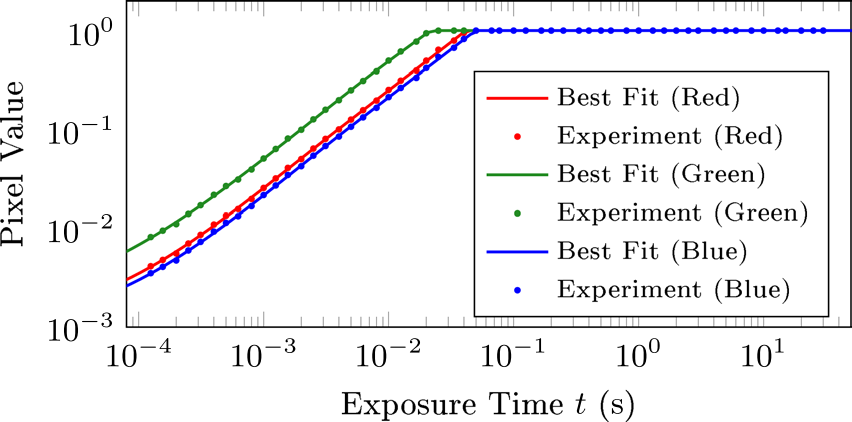
Empirically characterized best-fit response curves for each colour channel.

For incident light of intensity *I*_0_ and wavelength *λ*_0_, dimensionless relative radiant exposures can be mapped to pixel values $\zeta \in \mathrm{[0,}\,\mathrm{1]}$ via the sensor response function ${\mathscr{Z}}$ as follows:

7 $$\zeta ={\mathscr{Z}}({I}_{0}t/{\varphi }_{0}({\lambda }_{0})),$$

where *t* is the exposure time. The physical validity of this mapping is detailed in Supplementary Information B. The trichromatic approach adopted therefore requires only empirical determination of ${\mathscr{Z}}$ and ${\varphi }_{0}$ for each colour channel. Figure 4 shows the individual response curves for each colour channel obtained by capturing images of the object at different exposure times and averaging the channel-specific pixel value within a fixed predefined region on the image. The nonlinearity at low exposure times is attributed to semiconductor non-idealities. The peak wavelengths of each channel (600 nm, 530 nm, and 450 nm for R, G and B respectively) were estimated based on existing quantum efficiency curves of similar camera models^34^.

## Model Verification

To demonstrate the accuracy of the presented computational method, sets of theoretical predictions are compared against experimental measurements. The benchmark experiment setup is first described, followed by comparisons between experimental results and theoretical predictions.

### Experimental Setup

The characterization of the imaging device used has previously been detailed. The object in the benchmark experiment comprised a 20W LED floodlight with 5000 K colour temperature, over which a diffuser film was placed. A circular mask of diameter 10.5 mm was then mounted to create a uniform circular light source. The distance from the object to the imaging sensor plane was constant at 100.0 ± 0.1 cm. All experiments were conducted in a dark room to minimize ambient light pollution of images. The object occupied approximately 10% of the captured image area, and was axially aligned to ensure it remained within the isoplanatic patch.

The post-processing applied to these experimental images and theoretical predictions were identical. In particular, an RGGB Bayer pattern was used, and a white balance of [R, G, B] = [2, 1, 2] was applied to account for the ratio of red-, green- and blue-channel pixels on the imaging sensor. Conversion to geometrical scale was performed using a pixel size of 3.917 × 3.917 μm^35^. A gamma compression was also applied in accordance with the sRGB standard (see Supplementary Information A). No additional scaling was performed; the accuracy of the calculated magnification can thus be verified.

### Results

Corresponding theoretical predictions were computed using the presented method. Matrix sizes of *m* = *n* = 3750 were adopted for sufficient null padding, followed by a cropping to 1250 × 1250 px to enhance visual clarity.

**Figure 5:**
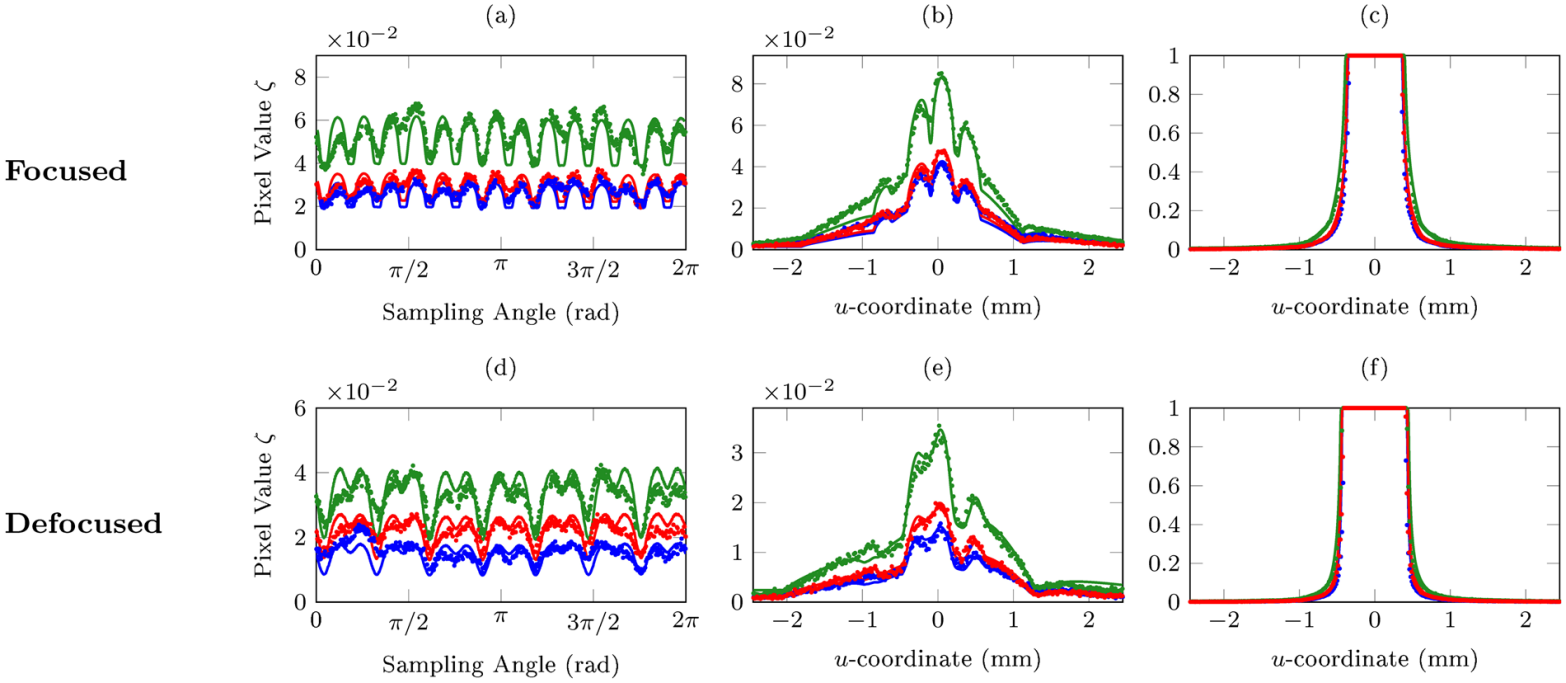
Image intensity profiles sampled along different paths, for both focused and defocused configurations: (**a**/**d**) circle of radius 0.335 *u*_max_ centered at the origin, (**b**) horizontal line 0.35 *v*_max_ from the top, (**e**) horizontal line 0.3 *v*_max_ from the top, and (**c**/**f**) horizontal line *v*^max^ from the top. *u*_max_ and *v*_max_ respectively denote half the spatial width and height of the cropped images. Lines and dots represent theoretical predictions and experimental measurements respectively; their colours represent the three colour channels (red, green, blue). The uncertainties in *ζ* and *u*-coordinate are approximately 7 × 10^−5^ and 3.917 μm respectively for all plots.

**Figure 6:**
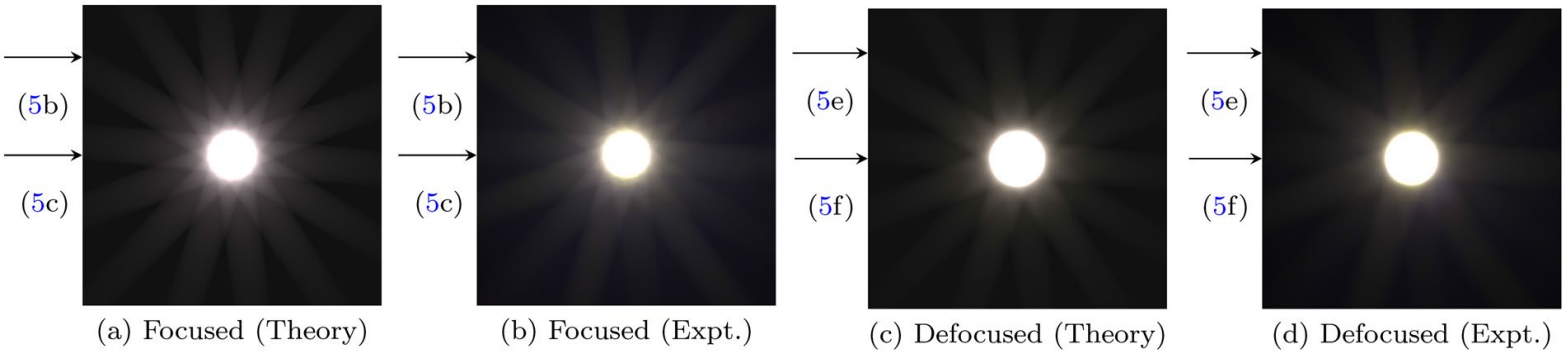
Comparison of theoretical image predictions and recorded experiment images, for both focused and defocused scenarios. All images are adjusted to sRGB colour space. Calculated MSE and SSIM metrics for these images are presented in Table 1. The horizontal arrows along the left side of each figure indicate the level of the cross-sections sampled in Fig. 5.

We compare theoretical predictions and experimental results for both a focused, diffraction-limited image as well as a severely defocused image. For each set of comparisons, image intensity profiles are plotted with circular as well as cross-sectional sampling (Fig. 5). Comparisons between rendered sRGB colour images are also presented (Fig. 6), for which the Mean Square Error (MSE)^36^ and Structural Similarity Index (SSIM)^37^ are used as quantitative measures for the accuracy of the predictions.

#### Focused Images

The focused configuration comprises *r*_o_ = *r*_f_ = 100.0 cm, *t* = 2.5 s, and a *f*/22 aperture. Excellent agreement on image intensity profiles can be observed in Fig. 5(a–c). Clearly noticeable in Fig. 5(a) is the number of distinct diffraction spikes (14), twice that of the number of edges on the polygonal aperture (7). Indeed, for even-sided apertures, the number of observable diffraction spikes will typically be identical to the number of aperture edges; whereas for odd-sided apertures, the number of diffraction spikes will be *twice* the aperture edge count. A qualitative explanation is presented in Supplementary Information A.

#### Defocused Images

The defocused configuration comprises *r*_o_ = 100.0 cm, *r*_f_ = 45.0 cm, *t* = 1.6 s, and a *f*/22 aperture, corresponding to a 4.78 mm defocus. Excellent accuracy of theoretical predictions can be observed in Fig. 5(d–f). A comparison between Fig. 5(a) and 5(d) makes explicit the effects of defocus on the starburst effect, where for similar sampling radii, the inner “spikes” are blurred out and merge with the larger “rays” emanating from the centre of the imaged light source. This effect is well visualized when comparing the rendered focused and defocused images in Fig. 6. Clearly, a good overall agreement has been achieved.

#### Colour Images

Figure 6 compares the final predicted colour images from the computational method with experiments, for both the focused and defocused imaging system configuration. A slight alternation in the intensity of the diffraction spikes is visible in Fig. 6(b), suggesting non-idealities in the MTF of the lens system used. Excellent colour agreement is evident for both focused and defocused images. In addition, excellent results are obtained for the MSE (<0.1%) and SSIM (>95%) comparison tests, detailed in Table 1.

**Table 1:** Calculated MSE and SSIM for focused and defocused comparisons.

	Focused		Defocused	
	MSE (×10^−3^)	SSIM	MSE (×10^−3^)	SSIM
R	0.311	0.965	0.478	0.951
G	0.270	0.950	0.381	0.960
B	0.868	0.973	0.676	0.966
B/W	0.304	0.979	0.403	0.968

Grayscale images were calculated from truecolour RGB images (Fig. 2) with weighting factors of [0.2989, 0.5870, 0.1140] for [R, G, B] channels respectively and then gamma compressed, as described by Equation (A.1) of the Supplementary Information. The low MSE (<0.1%) and high SSIM metrics (>95%) indicate good agreement between theoretical predictions and experiment data.

## Conclusion

This paper has discussed a rigorous framework for calculating the image of a starburst-affected scene viewed through an imaging system with arbitrary focus and aperture geometry, based fundamentally on a physically-valid Fourier optics formulation. Following the computation of channel-specific pixel values via a numerical double-transform method, we also propose a post-processing pipeline accommodating various image adjustments standard in modern imaging equipment. The final result is a direct analogue of images captured by imaging apparatus, inclusive of light transport effects within the apparatus, demosiacing mechanisms on the imaging sensor, and implicit programmatic image adjustments. Such comprehensiveness represents a key advancement over existing literature.

Notably, excellent agreement between predictions and real-world experimental measurements were observed, for both focused and defocused configurations. These benchmark results indicate outstanding accuracy of our computational method. The trichromatic approach enables the prediction of blurred colour images unachievable with existing monochromatic, unaberrated methods; the required characterization of light sources with unknown spectral power distributions is also greatly simplified, a notable advantage over full polychromatic approaches. Our study has also presented key methods for the characterization of optical parameters of imaging systems, including edge-detection techniques for pupil geometry determination and the measurement of sensor response curves—these methods can be utilized to characterize other systems, on which computational predictions can then be made.

The presented results are of great relevance to the modelling and reduction of diffraction spikes in telescope and telemetry acquisition systems, critical for data accuracy^1–3,9^; the Bahtinov mask-facilitated automated focusing of telescopes may also be improved, and spider-diffraction phenomena typically encountered in reflecting telescopes may also be optimized^16^. The framework may also be applied for the accurate generation of starburst visual effects in photorealistic computer graphics and the computation of diffraction spikes observed by the human eye, taking into account aberration effects^38,39^. Extension of the computational framework to model x-ray diffraction^40–42^ and complex diffraction phenomena^43–45^ is also plausible, due to its mathematical and structural generality.

## Electronic supplementary material

Supplementary Information

## Data Availability

The datasets are available from the corresponding author on reasonable request.
