# Supplementary material for: Computational Imaging Prediction of Starburst-Effect Diffraction Spikes
Source: Sci Rep. 2018 Nov 16;8:16919. doi: 10.1038/s41598-018-34400-z (PMC6240111; doi:10.1038/s41598-018-34400-z)
Supplement: Supplementary file 1 — Supplementary Information [file 41598_2018_34400_MOESM1_ESM.pdf]

Computational Imaging Prediction of Starburst-Effect Diffraction Spikes

Supplementary Information

Markus Lendermann[1], Joel Shi Quan Tan[2], Jin Ming Koh[3], and Kang Hao Cheong[3,*]

[1]National University of Singapore High School of Mathematics and Science, 20 Clementi Avenue 1, S129957, Singapore, Singapore
[2]Yong Loo Lin School of Medicine, National University of Singapore, S119228, Singapore, Singapore
[3]Engineering Cluster, Singapore Institute of Technology, 10 Dover Drive, S138683, Singapore, Singapore
[*]Corresponding Author: Kanghao.Cheong@SingaporeTech.edu.sg

Supplementary Information A: Optical Theory

A.1 Path-Length Error Calculation

The geometry considered for the calculation of the aberrational phase shift $kW(x,y)$ is illustrated in Figure A.1.

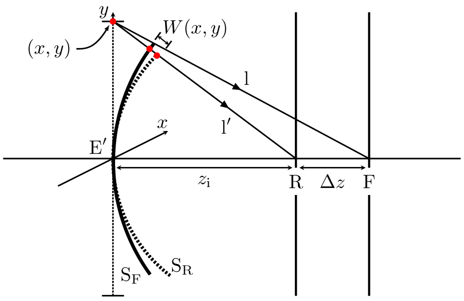

Figure A.1: An extension to the right half of Figure 1. F: in-focus image plane. $W(x,y)$ is the optical path-length accumulated between the reference spheres $\mathrm{S_F}$ and $\mathrm{S_R}$ (centered at the origins of F and R respectively) by the ideal ray l′ when traced backward from the ideal image point on R to the point (x,y) within the exit pupil E′.

A.2 Aperture Edge-Spike Ratio

For even-sided apertures, the number of observable diffraction spikes will typically be identical to the number of aperture edges; for odd-sided apertures, the number of diffraction spikes will be *twice* the aperture edge count. This can be qualitatively understood by considering that the aperture edges are the most significant contributors to diffractive effects. At each edge, diffraction spikes emanate in both normal directions—on an even-sided aperture, there is an overlap of diffraction spikes emanating from opposite edges, but such overlaps do not occur on odd-sided apertures, therefore leading to the differing spike-edge ratios.

A.3 Gamma Correction (sRGB)

The processing pipeline applied to experimental and theoretical results entailed a gamma compression on the linear sRGB images in accordance with the sRGB standard, given by

$$C_{\mathrm{srgb}} = \begin{cases} 12.92 C_{\mathrm{linear}} & , C_{\mathrm{linear}} \leq a \\ 1.055 C_{\mathrm{linear}}^{1/2.4} - 0.055 & , C_{\mathrm{linear}} > a, \end{cases} \tag{A.1}$$

where $a = 0.0031308$, and C is R, G, or B.

Supplementary Information B: Imaging System Characterization

B.1 Lens System Data

Table B.1 presents the pupil and principal plane positions of the examined AF Nikkor 50mm $f/1.8$D prime lens as extracted from existing literature [1].

E (mm)	E' (mm)	P (mm)	P' (mm)
48.8 ± 0.1	54.5 ± 0.1	48.5 ± 0.1	51.5 ± 0.1

Table B.1: Distances measured from sensor plane. E, E′, P and P′ denote the entrance pupil, exit pupil, front principal plane, and rear principal plane respectively.

B.2 Focusing Offset Calculations

For an object located at a distance r_o from the imaging sensor plane and a focusing distance of r_f, it can be deduced from Equation (6) that

$$\delta z = -\frac{1}{2}\left(2f + \gamma + \sqrt{4f\gamma + \gamma^2}\right), \tag{B.1}$$

$$\Delta z = \frac{(r_\mathrm{f} - r_\mathrm{o})\left(\sqrt{4f\gamma + \gamma^2} + 2f + \gamma\right)}{-\sqrt{4f\gamma + \gamma^2} + 2f + r_\mathrm{f} - 2r_\mathrm{o} + z_\mathrm{p}}, \tag{B.2}$$

where $\gamma = z_\mathrm{p} - r_\mathrm{f}$, and Δz is as defined in Figure A.1. The latter can then be used in Equation (3) for the calculation of path-length error.

B.3 Sensor Response Function

In an ideal linear sensor, the recorded pixel value ζ is proportional to the charge liberated at the sensor element, until saturation where $\zeta = 1$.

$$\zeta \propto \int_0^\infty \chi(\lambda) N_\nu(\lambda)\,\mathrm{d}\lambda = \int_0^\infty \chi(\lambda)\left(\frac{\mathcal{M}(\lambda)A\lambda}{\mathfrak{h}c}t\right)\mathrm{d}\lambda, \tag{B.3}$$

where $N_\nu(\lambda)$ is the number of photons absorbed per unit wavelength, $\mathcal{M}(\lambda)$ is the incident spectral power distribution (SPD), A is the pixel area, t is the exposure time, $\mathfrak{h}$ is Planck's constant, c is the speed of light, and $\chi(\lambda)$ is the quantum efficiency (QE) of the sensor. Keeping A and the light source identical, a linear response to exposure time can thus be expected for each colour channel, albeit with different gradients. In modern charge-coupled device (CCD) and complementary metal–oxide–semiconductor (CMOS) image sensors, this initially linear response is typically followed by a sharp plateau at the saturation point [2–5].

For monochromatic incident light of wavelength λ_0 and intensity I_0, Equation (B.3) reduces to

$$\zeta \propto \frac{A\chi(\lambda_0)\lambda_0}{\mathfrak{h}c}\left(\frac{I_0 t}{\phi_0(\lambda_0)}\right), \tag{B.4}$$

where $\phi_0(\lambda_0)$ is the wavelength-specific reference radiant exposure, defined as the minimum exposure necessary to achieve sensor saturation. As the coefficients are solely dependent on sensor properties, they can be encompassed in the sensor response function $\mathcal{Z}$ as introduced in Equation (5), yielding Equation (7) as presented.

It is thus apparent that unlike a complete polychromatic approach, the trichromatic approach adopted does not require knowlege of the complete SPD of the source and the QE of the imaging sensor. Instead, only empirical determination of $\mathcal{Z}$ and ϕ_0 for each colour channel is required.

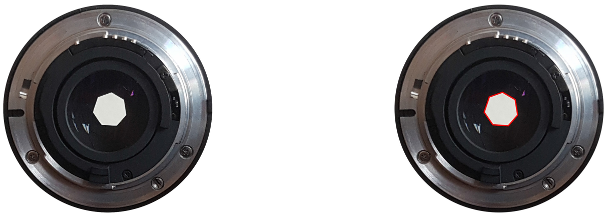

Figure B.1: Edge-detection of the exit pupil for an $f/5.6$ aperture. In the right image, in red: the edge-detected aperture shape.

B.4 Pupil Edge-Detection

A computational edge-detection method was used to characterize the size and geometry of the entrance and exit pupils. Figure B.1 presents the edge-detected pupil profile for the examined lens system.

References

[1] Wisniewski, J. S. Lens measurement table (2003). Data retrieved from Photography Resources, http://www.swissarmyfork.com/lens_table_1.htm, accessed 2012.

[2] Fossum, E. R. Cmos image sensors: electronic camera-on-a-chip. *IEEE Transactions on Electron Devices* **44**, 1689–1698 (1997).

[3] Fowler, B., Gamal, A. E., Yang, D. & Tian, H. Method for estimating quantum efficiency for cmos image sensors. *Proc. SPIE* **3301**, 178–185 (1998).

[4] Grossberg, M. D. & Nayar, S. K. Modeling the space of camera response functions. *IEEE Transactions on Pattern Analysis and Machine Intelligence* **26**, 1272–1282 (2004).

[5] Bigas, M., Cabruja, E., Forest, J. & Salvi, J. Review of cmos image sensors. *Microelectronics Journal* **37**, 433 – 451 (2006).
